# Laparoscopic Versus Open Hartmann Reversal: A Case-Control Study

**DOI:** 10.1155/2021/4547537

**Published:** 2021-01-23

**Authors:** Paolo Panaccio, Tommaso Grottola, Rossana Percario, Federico Selvaggi, Severino Cericola, Alfonso Lapergola, Maira Farrukh, Giuseppe Di Martino, Marco Ricciardiello, Pierluigi Di Sebastiano, Fabio Francesco Di Mola

**Affiliations:** ^1^Department of Medicine and Oral Sciences and Biotechnologies, Unit of General and Oncology Surgery, Casa Di Cura Pierangeli, University G. D'Annunzio, Chieti-Pescara, Italy; ^2^Department of Medicine and Oral Sciences and Biotechnologies, University G. D'Annunzio, Chieti-Pescara, Italy; ^3^Unit of General Surgery, Ospedale Floraspe Renzetti, Lanciano, Italy

## Abstract

**Background:**

Laparoscopic reversal of Hartmann's procedure (LHR) offers reduced morbidity compared with open Hartmann's reversal (OHR). The aim of this study is to compare the outcome of laparoscopic versus open Hartmann reversal.

**Materials and Methods:**

Thirty-four patients who underwent Hartmann reversal between January 2017 and July 2019 were evaluated. Patients underwent either LHR (*n* = 17) or OHR (*n* = 17). Variables such as numbers of patients, patient's age, sex, body mass index (BMI), comorbidities, ASA (American Society of Anesthesiology) score, indication for previous open sigmoid resection, mean operation time, rate of conversion to open surgery, length of hospital stay, mortality, and morbidity were retrospectively evaluated.

**Results:**

The two groups of patients were homogeneous for gender, age, body mass index, cause of primary surgery, time to reversal, and comorbidities. In 97% of the cases, HP was done by open surgery. Our data revealed no difference in mean operation time (LHR: 180.5 ± 35.1 vs. OHR: 225.2 ± 48.4) and morbidity rate, although, in OHR group, there were more severe complications. Less intraoperative blood loss (LHR: 100 ± 40 mL vs. OHR: 450 ± 125 mL; *p* value <0.001), shorter time to flatus (LHR: 2.4 days vs. OHR: 3.6 days; *p* value <0.021), and shorter hospitalization (LHR: 4.4 vs. OHR: 11.2 days; *p* value <0.001) were observed in the LHR group. Mortality rate was null in both groups. *Discussion*. LHR is feasible and safe even for patients who received a primary open Hartmann's procedure. We suggest careful patient's selection allowing LHR procedures to highly skilled laparoscopy surgeons.

## 1. Introduction

First described in 1923 by Henri Albert Hartmann for treatment or palliation of patients with rectosigmoid pathologies, the Hartmann procedure (HP) is a common major surgical procedure consisting in a sigmoidectomy followed by a terminal colostomy in the left iliac fossa and closure of the rectal stump. Nowadays, HP is used to treat emergent benign or malignant rectosigmoid diseases when the primary anastomosis is not possible [1, 2]. HP is a life-saving intervention which maintains an important role in urgent/emergent setting in frailty and septic patients [3]. The restoration of bowel continuity after HP defined as Hartmann reversal (HR) is a major procedure burdened by considerable postoperative morbidity and mortality rates. Closure rates are lower than 50% in most published series [4–10]. A large retrospective study showed that better chances of closure were found in nonneoplastic cases, younger age, male gender, and a low Charlson comorbidity index [11–13]. Despite HR still being generally performed by conventional open surgery, in recent years there has been increasing interest in the application of minimally invasive surgery (MIS) to reduce morbidity and mortality. Many authors have reported the safety and feasibility of laparoscopic HR (LHR) and comparative studies showed that the laparoscopic approach results in less blood loss, a shorter length of hospital stay, lower postoperative short-term morbidity, and less incisional hernia rate if compared to open Hartmann reversal (OHR) [11, 14]. However, the number of laparoscopic HR worldwide remains up to now low. A prospective observational multicenter study on all types of laparoscopic colorectal procedures reported that on 5,853 patients analyzed, the number of LHR reversals was less than 1% [15]. In addition, conversion to laparotomy can occur in a percentage of patients up to 15% and is usually due to dense adhesions or difficulty in identification of the rectal stump [16]. Therefore, if it is well accepted that HR is a feasible procedure, the high conversion rate must be seen in terms of a technically very demanding operation even in experienced hands. The aim of the study is to report our up-to-date experience with laparoscopic reversal of open Hartmann's operation compared to the open technique, encouraging an extensive use, when possible, of a minimally invasive approach.

## 2. Materials and Methods

A retrospective analysis of the prospectively collected database of all cases who underwent LHR and OHR intervention in our Units of General and Oncologic Surgery, in the period between January 2017 and July 2019 was conducted. Out of a total of 66 patients who underwent Hartmann procedure for complicated left-sided colonic disease in the 24 months preceding the period considered, 34 patients (52%) underwent Hartmann reversal between January 2017 and July 2019. Patients underwent either LHR (*n* = 17) or OHR (*n* = 17). Preoperative workup included physical examination, thoracoabdominal CT scan in oncological patients, blood count, and flexible endoscopy. No patient underwent mechanical bowel preparation. To all patients, we administered short-term antibiotic therapy with 2 g cephazolin and 500 mg metronidazole. All patients received thrombotic prophylaxis with low molecular weight heparin 12 h before surgery and then once daily until discharge according to the patient's comorbidities. Nasogastric tube was removed on awakening; the urinary catheter was placed after induction of general anaesthesia and removed on the first postoperative day. A perianastomotic penrose drain was routinely used. All patients followed an enhanced recovery after surgery (ERAS) protocol modified as we reported before [17]. Criteria for the discharge included absence of symptoms, flatus passage, and acceptable feeding. All laparoscopic and open procedures were performed or proctored by highly trained surgeons in colorectal surgery (FFDM, TG, and SC).

For comparison among the patients' group, variables such as numbers of patients, patient's age, sex, body mass index (BMI), comorbidities (e.g., hypertension, diabetes mellitus, and cardiovascular diseases), ASA (American Society of Anesthesiology) score, indication for previous open sigmoid resection, mean operation time, rate of conversion to open surgery, length of hospital stay, mortality, and morbidity were retrospectively evaluated. The main study outcome was 30-day mortality and overall morbidity (reoperation rate, anastomotic failure or stricture, postoperative ileus, and wound infection) according to Clavien–Dindo classification [18]. The choice of the surgical approach was based on the following principles: candidates for the laparoscopic restoration of intestinal continuity should be fit for surgery, with ASA scores I-III. In all other cases, an open approach has been chosen. Continuous variables were compared using the Mann–Whitney *U* test. Categorical variables were compared using the Chi-square test. All tests were considered statistically significant for a *p* value less than 0.005. Statistical analyses were performed using SPSS (IBM, SPSS Statistics, Version 20, Armonk, NY, USA) software.

### 2.1. Surgical Technique

**Figure 1:**
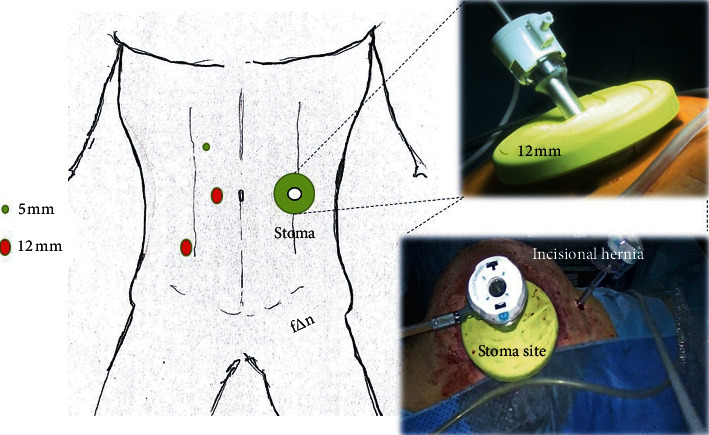
Trocar's positions with Alexis® wound protector.

**Figure 2:**
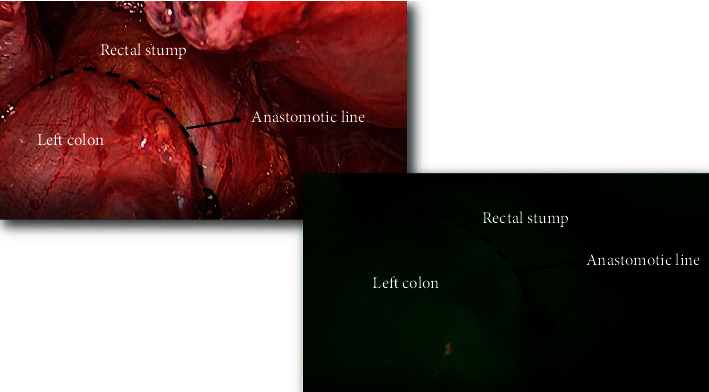
ICG-indocyanine green intraoperative angiography showing bowel perfusion.

OHR is a well-known procedure which includes the following steps: stoma's closure to minimize surgical field contamination, midline laparotomy, adhesiolysis in and around the stoma allowing visualization of the distal colon exiting the anterior abdominal wall, section of the proximal bowel/stoma over a circular anvil, rectal stump's identification and mobilization, splenic flexure mobilization, evaluation of suitability for a tensionless colorectal anastomosis and, finally, end-to-end or end-to-side colorectal anastomosis through Knight and Griffen technique. Hereby, we describe our technique in LHR. The operation was conducted positioning the patient in a 20–25-degree Trendelenburg with a slight rotation toward the right flank. The surgical operative steps were the following: mobilization of the stoma from the abdominal wall and its liberation from the rectum sheaths, excision of the colostomy from outside and mobilization of the bowel out of the abdomen, resection of the distal part of the stoma, and stapler anvil introduction in the proximal colon by purse-string suturing, returning of the bowel in the abdominal cavity, peritoneal sealing allowed by a “cap” (Alexis® laparoscopic system with Kii Fios First Entry, Applied Medical, Rancho Santa Margarita, CA), pneumoperitoneum through the Alexis® disposable, and placement of other three operative trocars in a standard position for a rectosigmoid laparoscopic resection as shown in Figure 1. One additional trocar was needed in two patients (13%) due to a difficult mobilization of the splenic flexure and severe bowel adhesions. Therefore, the laparoscopic phase proceeded with an extended laparoscopic inspection of the abdominal cavity, small and large bowel adhesiolysis, and rectal mobilization. A full release of the splenic flexure to achieve a tension-free anastomosis was done if requested and if not done during previous HP. A circular stapler was used to reestablish bowel continuity through an end-to-end anastomosis according to the Knight–Griffen technique. A routine ICG-indocyanine green intraoperative angiography was performed to assess the anastomosis perfusion as shown in Figure 2. Intraoperative proctosigmoidoscopy to assess anastomotic integrity was performed only in doubtful air-leak tests.

## 3. Results

Between January 2017 and July 2019, 34 consecutive reversal procedures were performed, 17 of which are laparoscopically (LHR group) and 17 open (OHR group). The two groups of patients were homogeneous for gender, age, BMI, cause of primary surgery, time to reversal, and comorbidities. In the LHR group, the male/female ratio was 8/9, with a mean age of 68.5 ± 12.2 years. In the OHR group, the male/female ratio was 7/10 with a mean age of 70 ± 9.4 years (*p* value = 0.784).

The mean BMI was 27.2 ± 7.3 kg/m^2^ and 24.5 ± 5.3 kg/m^2^ in the LHR group and in the OHR group, respectively (*p* value = 0.524). No differences in ASA score were observed between the groups. 33 patients had previously undergone an open Hartmann's procedure for obstructive or perforated rectosigmoid cancer or complicated acute diverticulitis meanwhile only one patient underwent a laparoscopic Hartmann due to acute diverticulitis with local peritonitis. The median interval time from the open emergency procedure to reversal was 158 days (range 88–335) in the LHR group and 209 (range 87–379) in the OHR group (*p* value = 0.185). Indications and patient's characteristics are summarized in Table 1. In the LHR group, all procedures were completed without conversion to the open technique. Additionally, no patient required a temporary colostomy or ileostomy. In the LHR group, 3 patients required an intraoperative resection of the rectal stump due to rectum stenosis (evaluated preoperatively with flexible endoscopy), 1 patient requested reresection of colorectal anastomosis due to impaired perfusion highlighted by ICG-indocyanine green intraoperative angiography. In two cases of the LHR group, patients had also a large median incisional hernia due to the previous surgery that was not treated during LHR but was done for a second time in both cases by laparoscopic intraperitoneal on-lay mesh (IPOM). One patient of the LHR underwent previously an open pancreaticoduodenectomy for a T2N0M0 papilla adenocarcinoma. Five patients (30%) in the LHR group and 2 patients (12%) in the OHR group, respectively, did not require a complete takedown of the splenic flexure and a good tension-free anastomosis was achieved just by mobilizing the left colon. Intraoperative proctosigmoidoscopy showed an average anastomotic distance from the anal verge of 8.2 ± 4.3 cm and 7.5 ± 3.3 in the LHR group and OHR group, respectively. The mean operative time was 180.5 ± 35.1 minutes in the LHR group and 225.2 ± 48.4 minutes in the OHR group (*p* value = 0.089). Less intraoperative blood loss (LHR:100 ± 40 mL vs. OHR: 450 ± 125 mL, *p* value <0.001), shorter time to flatus (LHR: 2.4 (range 1–5) vs. OHR: 3.6 (range 2–7) days, *p* value <0.021), and shorter hospitalization (LHR: 4.4 (range 3–11) vs. OHR: 11.2 days (range 6–33), *p* value <0.001) were observed in the LHR group.

Overall, postoperative complications were observed in 4 patients (3 wound infections, 1 postoperative ileus) in the LHR group and in 7 (4 wound infections, 2 postoperative ileus, and 1 anastomotic leakage) in OHR group, respectively, (*p* value <0.364). Notably, in the LHR group, there were only wounds infections related to the colostomy site closure. Moreover, in the LHR group, only Clavien–Dindo grade I-II complications were reported while in the OHR group one Clavien–Dindo grade III and two grade IV complications were observed. Readmission and 30-day mortality rate were null in both groups. Operative, postoperative, and morbidity data are detailed in Tables 2 and 3.

## 4. Discussion

HP is currently performed when it is necessary to contain the septic outbreak and local or systemic conditions do not allow primary anastomosis [19, 20]. HR is still a demanding procedure associated with morbidity ranging between 16 and 50% and a nonnegligible mortality reaching a 7% rate [2, 21]. Due to its perioperative risk and complication rates, up to 40–60% of patients never have a reversal [22–24]. According to that, in our experience, the rate of reversal was 52%. Besides that, there is no clear consensus on its appropriate timing. Fleming and Gillen as well as Roe et al. have suggested that a long time period between the initial surgery and reversal may lead to the shrinkage of the distal stump leading to a more difficult dissection and anastomosis [19, 25]. Aydin et al. studied 121 patients who underwent successful Hartmann's reversal and their results suggest that closure within 4 months was the reliable time to proceed [26]. Pearce et al. reviewed 145 patients who underwent Hartmann's reversal and found that a waiting period of 6 months was the safest for patients [27]. More interestingly, Keck et al. have evaluated 111 Hartmann's reversals reporting no difference in morbidity, mortality, or complication rates between those patients who had their takedown early (before 15 weeks) or late (after 15 weeks) [28]. Besides that, it seems that early reversal (<3 months) may lead to complications secondary to adhesions and residual inflammation; on the other end of the spectrum, it is thought that waiting too long may lead to difficulty in mobilizing and anastomosing the rectal stump. The significant rate of complications reported following reversal of a Hartmann's colostomy via laparotomy has led many groups to explore the feasibility of laparoscopically assisted reversal. To date, primary procedure (HP) is still preferably performed with an open approach as it is performed after urgent or emergent colonic resections due to suppurative or stercoraceous peritonitis, especially in ASA IV patients [9, 29, 30]. According to the current literature, patients who had undergone several abdominal surgeries and who had clinically relevant cardiovascular and pulmonary comorbidities usually received an OHR [31, 32]. Moreover, some authors state that a shorten rectal stump seems to be a common indication for OHR too [8, 33, 34]. Nevertheless, the laparoscopic restoring of colonic continuity after Hartmann's rectosigmoid resection, especially after the open primary approach, remains a challenging and not well-standardized procedure. Gorey et al. in 1993 have first reported a laparoscopically assisted stoma reversal concluding that the procedure might lead to a shorter hospital stay and increased patient acceptance [35]. However, different authors have documented many advantages over the same open surgery, particularly with regard to overall postoperative morbidity (wound infections and postoperative ventral hernia), length of hospital stay, and return to daily activities [11, 36–39]. Despite the fact that LHR is a procedure that does not belong to all surgeons, the use of laparoscopic reversal increased dramatically over time, from about zero in 2005 to more than 25% in 2014, quickly becoming a “must-have” in every colorectal surgeon “armamentarium” [23]. The difficult complete freeing of the splenic flexure, extensive adhesiolysis, and the identification and mobilization of the rectal stump are some of the causes of its slow acceptance from the surgical community. As a matter of fact, Jamali et al. have conducted a mail-in survey of 35 experienced laparoscopic colorectal surgeons, reporting the highest score of difficulty for a procedure involving splenic flexure release and rectal mobilization. Interestingly, in our experience, we think that the systematic use of the ICG-indocyanine green intraoperative angiography during laparoscopy might reduce the extensive use of splenic flexure's mobilization allowing a satisfying anastomosis. Hartmann reversal was felt to be one of the most difficult procedures emerging that minimally invasive Hartmann's takedown is best left for the advanced stages of colorectal surgeon's experience [40]. According to that, making one of the most difficult steps easier, during the open HP, we usually prefer to overclose the rectal stump with nonabsorbable stay stitches securing the rectal stump to the left pelvic wall in order to facilitate subsequent stump's localization, especially in the laparoscopic second step. To determine the feasibility of LHR, after stoma's mobilization, we usually perform diagnostic laparoscopy using the colostomy site as a first port and; then, we introduce ports to assess the severity of adhesion and to assess the rectal stump's viability. Occasionally, the introduction of the circular stapler into the rectum could help in the identification and mobilization of the rectal stump. The current literature reports a conversion rate to conventional surgery close to 20%, with significant variations among the earlier published studies and the more recent ones (range 0–20 percent) [15, 41, 42]. This difference seems to reflect a wider application of minimally invasive techniques and the increasing experience of a growing number of highly minimally -invasive skilled colorectal surgeons. In our LHR group, we experienced a zero-conversion rate probably because this is a procedure that we have used in our last stages of the learning curve. In our experience, LHR allows significantly shorter hospitalization, less intraoperative blood loss, less mean operative time, and quicker return of bowel function. Laparoscopic reversal leads to a reduction in complication rates, especially with regard to the most severe ones. LHR compared to OHR showed a lower rate of wound infections (SSI) although it did not reach statistical significance. In previous studies, it appears that the indications to minimally invasive surgery were selected based on the younger age and lesser comorbidities. In a recent literature review, Celentano and Giglio have demonstrated that the indication for the HP showed a trend toward more benign patients included in the laparoscopic group, and the interval time between the index Hartmann's procedure and its reversal was significantly shorter in the laparoscopic group with a trend toward a higher rate of temporary ileostomy in patients undergoing an open procedure. Moreover, when the first sigmoidectomy is performed for malignant disease, the interval between the HP and the reversal is longer and the procedure tends to be performed via an open approach [43]. In our series, LHR and OHR group are homogeneous, showing no statistically significant differences regarding neither the primary disease requiring HP nor comorbidities. This study was limited by its retrospective nature of comparing two nonrandomized groups. Despite the fact that the principal limitation of the present study is the limited sample size, we would like to underline that, in contrast with many case series present in the literature, almost all LHR's patients underwent a previous open HP and, in some cases, patients had also a large incisional hernia, which has not prevented a laparoscopic procedure. Therefore, there was a selection bias in the study, which may explain the significantly improved safety profile of the laparoscopic approach. Moreover, the decision regarding the surgical approach was based on a combination of patient performance status and surgeon's preference.

## 5. Conclusions

LHR appears to be safe and feasible compared to OHR and this agrees with recent studies. LHR achieves faster positive results in relation to OHR. Unfortunately, the lack of larger multicentric prospective and randomized study on LHR compared to OHR does not allow drawing standardized surgical procedures and objective conclusions. After that, many authors “speak loudly” that LHR is recommended as the first-line operative technique as it is associated with faster recovery and better outcomes when compared to OHR; we strongly suggest that LHR could be performed safely only in high-volume centers and by a well-trained laparoscopic team.

## Figures and Tables

**Table 1:** Patient's characteristics.

	LHR (*n* = 17)	OHR (*n* = 17)	*p* value
**Gender**, M/F (%M)	8/9 (47)	7/10 (41)	0.862^*a*^
**Age**, mean ± SD	68.5 ± 12.2	70 ± 9.4	0.784^*b*^
**BMI**, mean ± SD	27.2 ± 7.3	24.5 ± 5.3	0.524^*b*^

**Cause of HR**, *n* (%)			
Acute diverticulitis (Hinchey II)	1 (6)	1 (6)	0.570^*a*^
Acute diverticulitis (Hinchey III)	4 (24)	3 (18)	0.570^*a*^
Acute diverticulitis (Hinchey IV)	5 (29)	8 (47)	0.570^*a*^
Sigmoid colon obstruction	5 (29)	4 (24)	
Sigmoid ischaemia or anastomotic leak	2 (12)	1 (6)	
**Malignant disease**, *y* (*y*%)	9 (53)	8 (47)	0.706^*a*^
**Median time to reversal**, days (range)	158 (88–335)	209 (87–379)	0.185^*b*^

**Comorbidities**			
Diabetes mellitus	4 (24)	6 (35)	0.886^*a*^
Hypertension	8 (47)	8 (47)	0.503^*a*^
Cardiovascular diseases	3 (18)	5 (30)	0.761^*a*^

SD: standard deviation; BMI: body mass index (kg/m^2^); HR: Hartmann resection; ^*a*^chi-squared test; ^*b*^Mann–Whitney *U* test.

**Table 2:** Operative and morbidity data.

	LHR (*n* = 17)	OHR (*n* = 17)	*p* value
**Operative time**, mean ± SD	180.5 ± 35.1	225.2 ± 48.4	0.089^*a*^
**Intraoperative blood loss**, mean ± SD	100 ± 40	450 ± 125	<0.001^*a*^
**Hospitalization**, days (range)	4.4 (3–11)	11.2 (6–33)	<0.001^*a*^
**Time to first flatus**, days	2.4 (1–4)	3.6 (2–5)	0.021^*a*^
**Postoperative complications**, *n* (%)	4 (24)	7 (41)	0.364^*b*^
Anastomotic failure	0 (0)	1 (6)	0.998
SSI (superficial)	3 (17)	4 (23)	0.777
Postoperative ileus	1 (6)	2 (12)	0.909

SD: standard deviation; SSI: surgical site infection (superficial; ^*a*^Mann–Whitney *U* test; ^*b*^chi-squared test.

**Table 3:** Postoperative complications according to the Clavien–Dindo classification.

	LHR (*n* = 17)	OHR (*n* = 17)	*p* value^*∗*^
			0.386
**Grade I** (%)	2 (12)	3 (18)	
**Grade II** (%)	1 (6)	1 (6)	
**Grades IIIa**, **IIIB**, **IV**, **V** (%)	0 (0)	3 (18)	

Cited as [18] Clavien et al., “the Clavien–Dindo classification of surgical complications,” Ann. Surg., vol. 250, no. 2, pp. 187–196, 2009;  ^*∗*^chi-square test.

## Data Availability

The nominal and ordinal data used to support the findings of this study are available from the corresponding author upon request.

## References

[1] (1984). Classic articles in colonic and rectal surgery. Henri hartmann 1860–1952. New procedure for removal of cancers of the distal part of the pelvic colon. *Diseases of the Colon and Rectum*.

[2] Roque-Castellano C., Marchena-Gomez J., Hemmersbach-Miller M. (2007). Analysis of the factors related to the decision of restoring intestinal continuity after Hartmann’s procedure. *International Journal of Colorectal Disease*.

[3] Barbieux J., Plumereau F., Hamy A. (2016). Current indications for the Hartmann procedure. *Journal of Visceral Surgery*.

[4] Hallam S., Mothe B., Tirumulaju R. (2018). Hartmann’s procedure, reversal and rate of stoma-free survival. *The Annals of The Royal College of Surgeons of England*.

[5] Siddiqui M. R., Sajid M. S., Baig M. K. (2010). Open vs laparoscopic approach for reversal of Hartmann’s procedure: a systematic review. *Colorectal Disease: The Official Journal of the Association of Coloproctology of Great Britain and Ireland*.

[6] Haughn C., Ju B., Uchal M. (2008). Complication rates after Hartmann’s reversal: open vs. laparoscopic approach. *Diseases of the Colon and Rectum*.

[7] Cellini C., Deeb A. P., Sharma A. (2013). Association between operative approach and complications in patients undergoing Hartmann’s reversal. *British Journal of Surgery*.

[8] Maitra R. K., Pinkney T. D., Mohiuddin M. K., Maxwell-Armstrong C. A., Williams J. P., Acheson A. G. (2013). Should laparoscopic reversal of Hartmann’s procedure be the first line approach in all patients?. *International Journal of Surgery*.

[9] Kwak H. D., Kim J., Kang D. W., Baek S.-J., Kwak J. M., Kim S.-H. (2018). Hartmann’s reversal: a comparative study between laparoscopic and open approaches. *ANZ Journal of Surgery*.

[10] Holland J. C., Winter D. C., Richardson D. (2002). Laparoscopically assisted reversal of Hartmann’s procedure revisited. *Surgical Laparoscopy, Endoscopy & Percutaneous Techniques*.

[11] Sherman K., Wexner S. (2017). Considerations in stoma reversal. *Clinics in Colon and Rectal Surgery*.

[12] Seetharam S., Paige J., Horgan P. G. (2003). Impact of socioeconomic deprivation and primary pathology on rate of reversal of Hartmann’s procedure. *The American Journal of Surgery*.

[13] David G. G., Al-Sarira A. A., Willmott S. (2009). Use of Hartmann’s procedure in England. *Colorectal Disease*.

[14] Lucchetta A., De Manzini N. (2016). Laparoscopic reversal of Hartmann procedure: is it safe and feasible?. *Updates in Surgery*.

[15] Scheidbach H., Lippert H. (2006). Laparoscopic approach for Hartmann reversal procedures. *Journal of Minimal Access Surgery*.

[16] Rosen M., Cobb W., Kercher K., Heniford B. (2006). Laparoscopic versus open colostomy reversal: a comparative analysis. *Journal of Gastrointestinal Surgery*.

[17] Di Sebastiano P., Festa L., De Bonis A. (2011). A modified fast-track program for pancreatic surgery: a prospective single-center experience. *Langenbeck’s Archives of Surgery*.

[18] Clavien P. A., Barkun J., de Oliveira M. L. (2009). The clavien-dindo classification of surgical complications. *Annals of Surgery*.

[19] Fleming F. J., Gillen P. (2009). Reversal of Hartmann’s procedure following acute diverticulitis: is timing everything?. *International Journal of Colorectal Disease*.

[20] Breitenstein S., Kraus A., Hahnloser D., Decurtins M., Clavien P.-A., Demartines N. (2007). Emergency left colon resection for acute perforation. Primary anastomosis or Hartmann’s procedure? A case-matched control study. *World Journal of Surgery*.

[21] Bell C., Asolati M., Hamilton E. (2005). A comparison of complications associated with colostomy reversal versus ileostomy reversal. *The American Journal of Surgery*.

[22] van de Wall B. J. M., Draaisma W. A., Schouten E. S., Broeders I. A. M. J., Consten E. C. J. (2010). Conventional and laparoscopic reversal of the Hartmann procedure: a review of literature. *Journal of Gastrointestinal Surgery*.

[23] Pei K. Y., Davis K. A., Zhang Y. (2008). Assessing trends in laparoscopic colostomy reversal and evaluating outcomes when compared to open procedures. *Surgical Endoscopy*.

[24] Toro A., Ardiri A., Mannino M. (2014). Laparoscopic reversal of Hartmann’s procedure: state of the art 20 years after the first reported case. *Gastroenterology Research and Practice*.

[25] Roe A. M., Prabhu S., Ali A., Brown C., Brodribb A. J. M. (1991). Reversal of Hartmann’s procedure: timing and operative technique. *British Journal of Surgery*.

[26] Aydin N. H., Remzi F. H., Tekkis P. P., Fazio V. W. (2005). Hartmann’s reversal is associated with high postoperative adverse events. *Diseases of the Colon & Rectum*.

[27] Pearce N. W., Scott S. D., Karran S. J. (1992). Timing and method of reversal of Hartmann’s procedure. *British Journal of Surgery*.

[28] Keck J. O., Collopy B. T., Ryan P. J., Fink R., Mackay J. R., Woods R. J. (1994). Reversal of Hartmannʼs procedure:. *Diseases of the Colon & Rectum*.

[29] Fiscon V., Portale G., Mazzeo A., Migliorini G., Frigo F. (2014). Laparoscopic reversal of Hartmann’s procedure. *Updates in Surgery*.

[30] Park W., Park W. C., Kim K. Y., Lee S. Y. (2018). Efficacy and safety of laparoscopic Hartmann colostomy reversal. *Annals of Coloproctology*.

[31] Onder A., Gorgun E., Costedio M. (2016). Comparison of short-term outcomes after laparoscopic versus open hartmann reversal. *Surgical Laparoscopy, Endoscopy & Percutaneous Techniques*.

[32] Giuseppe R., Nicolò ID F., Serafino M. (2019). Laparoscopic reversal of Hartmann’s procedure: a single‐center experience. *Asian Journal of Endoscopic Surgery*.

[33] Zimmermann M., Hoffmann M., Laubert T. (2013). Laparoscopic versus open reversal of a Hartmann procedure: a single-center study. *World Journal of Surgery*.

[34] Schmelzer T. M., Mostafa G., Norton H. J. (2007). Reversal of Hartmann’s procedure: a high-risk operation?. *Surgery*.

[35] Gorey T. F., O’connell P. R., Waldron D. (1993). Laparoscopically assisted reversal of Hartmann’s procedure. *British Journal of Surgery*.

[36] Royo-Aznar A., Moro-Valdezate D., Martín-Arévalo J. (2018). Reversal of Hartmann’s procedure: a single-centre experience of 533 consecutive cases. *Colorectal Disease*.

[37] Mazeh H., Greenstein A. J., Swedish K. (2009). Laparoscopic and open reversal of Hartmann’s procedure-a comparative retrospective analysis. *Surgical Endoscopy*.

[38] Wigmore S. J., Duthie G. S., Young I. E., Spalding E. M., Rainey J. B. (1995). Restoration of intestinal continuity following Hartmann’s procedure: the Lothian experience 1987–1992. *British Journal of Surgery*.

[39] de’Angelis N., Brunetti F., Memeo R. (2013). Comparison between open and laparoscopic reversal of Hartmann’s procedure for diverticulitis. *World Journal of Gastrointestinal Surgery*.

[40] Jamali F. R., Soweid A. M., Dimassi H. (2008). Evaluating the degree of difficulty of laparoscopic colorectal surgery. *Archives of Surgery*.

[41] Ballian N., Zarebczan B., Munoz A. (2009). Routine evaluation of the distal colon remnant before Hartmann’s reversal is not necessary in asymptomatic patients. *Journal of Gastrointestinal Surgery: Official Journal of the Society for Surgery of the Alimentary Tract*.

[42] Brathwaite S., Latchana N., Esemuede I., Harzman A., Husain S. (2017). Risk factors for surgical site infection in open and laparoscopic Hartmann closure. *Surgical Laparoscopy, Endoscopy & Percutaneous Techniques*.

[43] Celentano V., Giglio M. C. (2018). Case selection for laparoscopic reversal of Hartmann’s procedure. *Journal of Laparoendoscopic & Advanced Surgical Techniques*.

